# The overexpression of MDM4: an effective and novel predictor of gastric adenocarcinoma lymph node metastasis

**DOI:** 10.18632/oncotarget.11971

**Published:** 2016-09-12

**Authors:** Junjie Bao, Abiyasi Nanding, Haibin Song, Rui Xu, Guofan Qu, Yingwei Xue

**Affiliations:** ^1^ Department of Gastroenterologic Surgery, Harbin Medical University Cancer Hospital, Harbin, China; ^2^ Department of Orthopedics, Harbin Medical University Cancer Hospital, Harbin, China; ^3^ Department of Pathology, Harbin Medical University Cancer Hospital, Harbin, China; ^4^ Department of Dermatology, Harbin Children's Hospital, Harbin, China

**Keywords:** MDM4, gastric adenocarcinoma, clinicopathological parameters, lymph node metastasis

## Abstract

**Background:**

MDM4 is the important negative regulator of the tumor suppressor protein p53, which is overexpressed in various human cancers. This study evaluates the MDM4 expression in patients with gastric adenocarcinoma (GTAC) at the mRNA and protein levels and examines relationships among MDM4 expression, clinicopathological features, and prognosis.

**Results:**

The qRT-PCR and the Western blot analysis showed that the MDM4 expression level was high in GTACN^+^ but not in GTACN^−^. The high expression level of MDM4 was significantly associated with age (*P* = 0.047), lymph node metastasis (LNM) (*P* < 0.001), pathological stage (*P* < 0.001), differentiation status (*P* = 0.001), and preoperative serum CA19-9 level (*P* < 0.001). Moreover, the survival analysis showed that Borrmann type, depth of invasion, LNM, and preoperative serum CA19-9 level were independent prognostic factors. The univariate analysis revealed that MDM4 expression influenced GTAC prognosis. Furthermore, the influence of overall prognosis relies on whether or not the high MDM4 expression level could lead to LNM.

**Materials and Methods:**

We investigated MDM4 expression in primary GTAC and paired normal gastric tissues (30 pairs) through qRT-PCR and Western blot analyses. We also performed immunohistochemistry analysis on 336 paraffin-embedded GTAC specimens and 33 matched normal specimens.

**Conclusions:**

MDM4 expression may result in LMN of GTAC. High MDM4 expression levels are associated with LMN of GTAC and influence the prognosis of patients with GTAC.

## INTRODUCTION

Gastric cancer, a common malignant tumor, remains a major cause of death worldwide despite its decreased incidence over past decades [1, 2]. About 90% to 95% of Gastric cancers are adenocarcinomas. Lymph node metastasis (LNM) is an important prognostic factor in gastric cancer [3, 4]. Among patients with R0 resection for gastric cancer, LN status is the most important independent prognostic factor, followed by pT category, surgical complication, and distant metastasis [5]. No accurate and reliable judgment of preoperative LNM has been established for patients with GTAC. Enhanced computed tomography (CT) is routinely used to evaluate LNM in gastric cancer, with a sensitivity of 80.0% and a specificity of 77.8% based on the size of LN [6]. However, the sensitivity of CT imaging is only 6.1%, and metastasized lymph nodes are smaller than 5 mm [7]. Accurate knowledge of the LN status helps predict prognosis and plan the extent of lymphadenectomy. Therefore, objective markers must be developed to identify LNM in GC.

The MDM4 gene encodes a nuclear protein that contains a p53-binding domain at the N-terminus and a RING finger domain at the C-terminus. MDM4 is overexpressed in various human cancers [8, 9]. MDM4 inhibits p53 by binding to its transcriptional activation domain. In addition, MDM4 interacts with the MDM2 protein via the RING finger domain and inhibits the degradation of the latter [10]. We collected microarray data of 186 GTAC cases and 33 normal gastric tissue cases from the Cancer Genome Atlas database. The bioinformatic screening showed that MDM4 expression is associated with LNM of GTAC (GTACN^+^). Similar approaches have been widely applied in previous studies [11, 12]. The screening results need to be examined by further investigations. Therefore, the purpose of this study is to verify the relationship between MDM4 and LNM in GTAC.

## RESULTS

### Overexpression of MDM4 in fresh GTACN^+^ tissues

**Figure 1:**
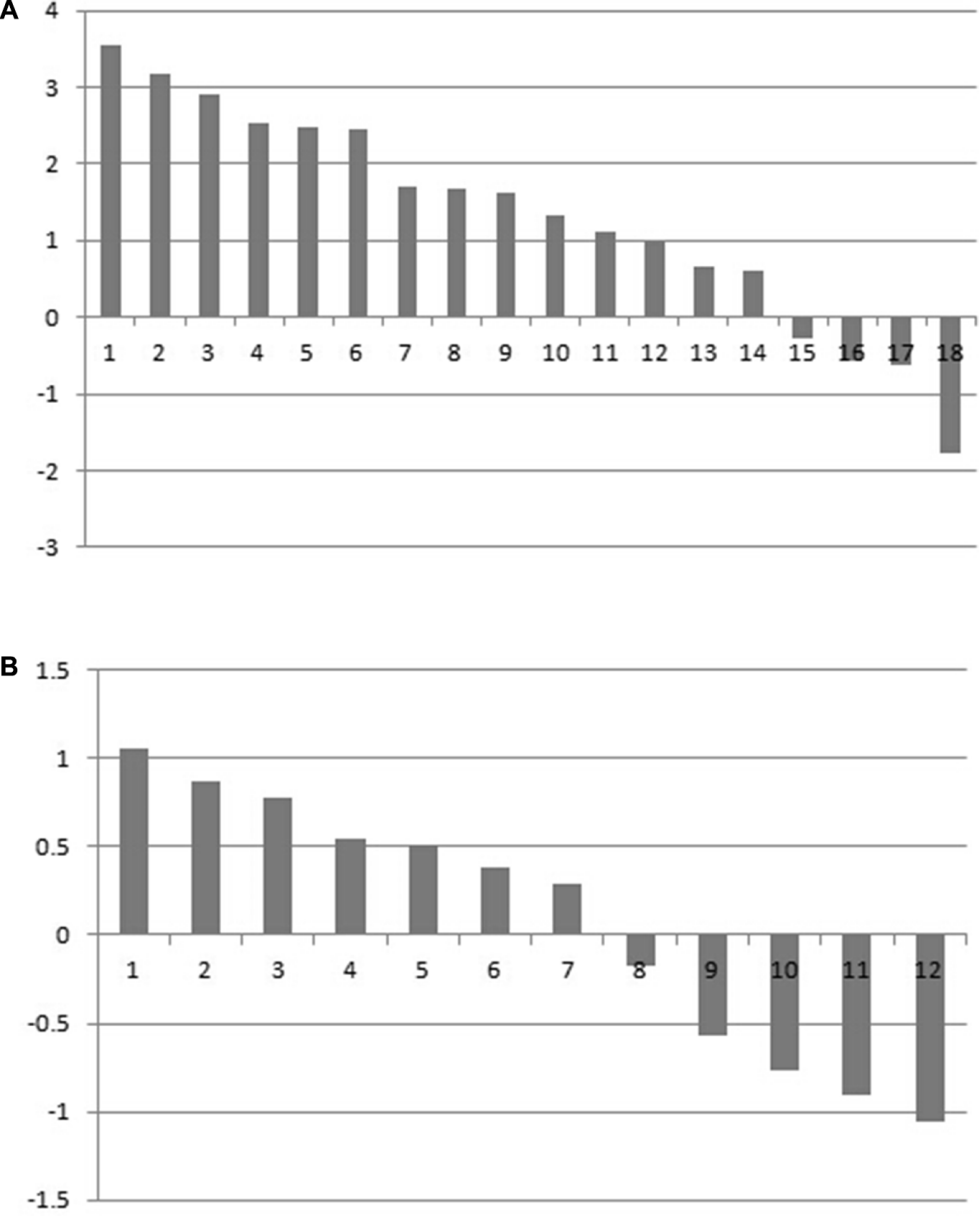
(**A**) MDM4 mRNA expression in GTAC tissues with positive lymph node metastasis and corresponding normal tissues. (**B**) MDM4 mRNA expression in GTAC tissues with negative lymph node metastasis and corresponding normal tissues.

The result of the bioinformatics analysis was verified by the first analyzing MDM4 expression in 30 pairs of GTAC and matched adjacent normal tissues at the mRNA level. 18 patients with GTAC exhibited positive LNM (GTACN^+^). qRT-PCR results showed that 14/18 GTACN^+^ displayed high MDM4 expression level (Figure 1A), which differed between the two groups (*t* = 2.695, *P* = 0.015). 12 patients with GTAC showed negative LNM (GTACN^−^). 7/12 GTACN^+^ displayed high MDM4 expression level (Figure 1B), which differed between the two groups (*t* = 0.355, *P* = 0.729).

**Figure 2:**
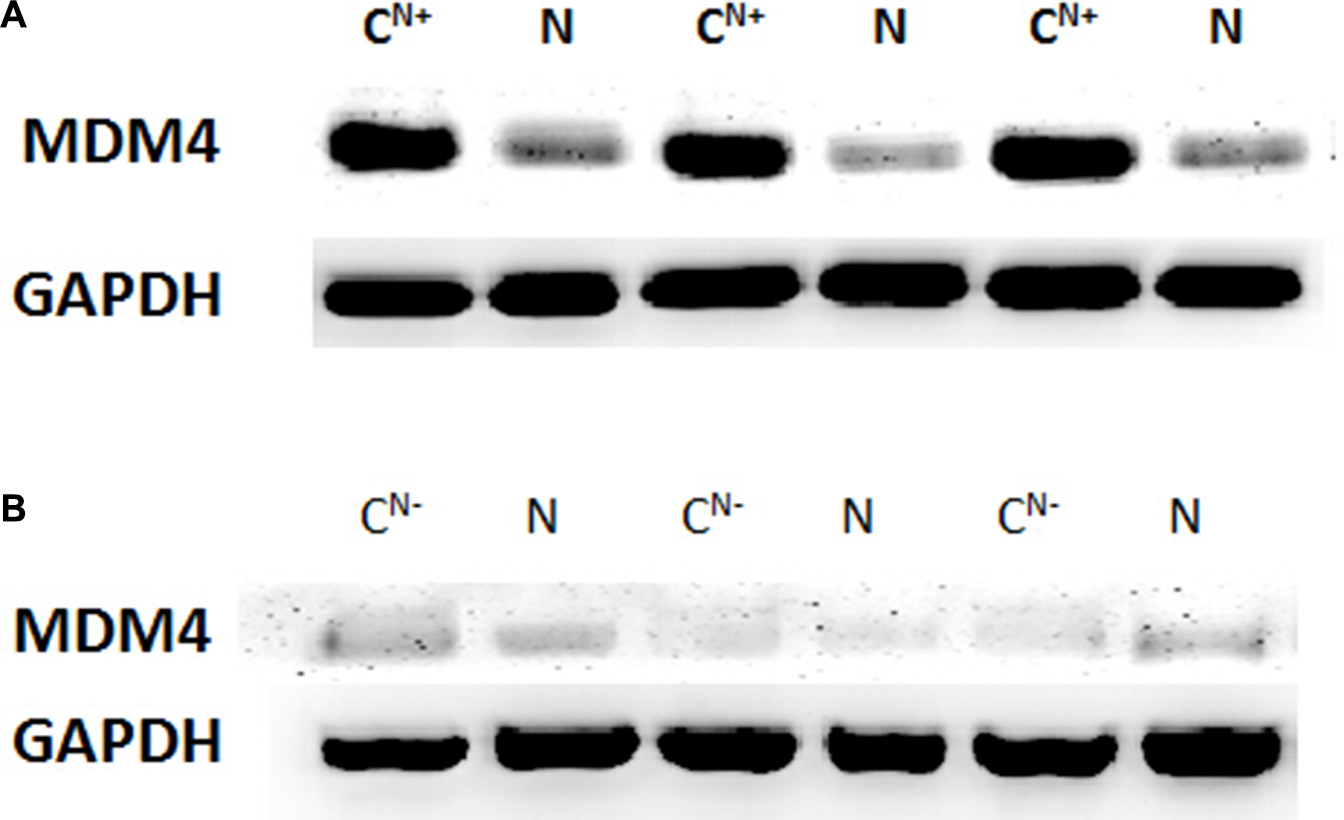
(**A**) The difference of MDM4 expression between GTAC tissues with positive lymph node metastasis and normal gastric tissues. (**B**) The difference of MDM4 expression between GTAC tissues with negative lymph node metastasis and normal gastric tissues.

The Western blot analysis was employed to determine the expression status in 30 pairs of fresh GTAC and adjacent normal gastric tissues and protein expression of MDM4 in GTAC. The results showed that MDM4 expression is high in GTACN^+^ (10/18) (Figure 2A) but low in GTACN^−^ (5/12) (Figure 2B). The result of the Western blot analysis was conformed with that of qRT-PCR in the 30 pairs of fresh GTAC tissues.

### Overexpression of MDM4 is associated with the clinicopathological characteristics of patients with GTAC

**Figure 3:**
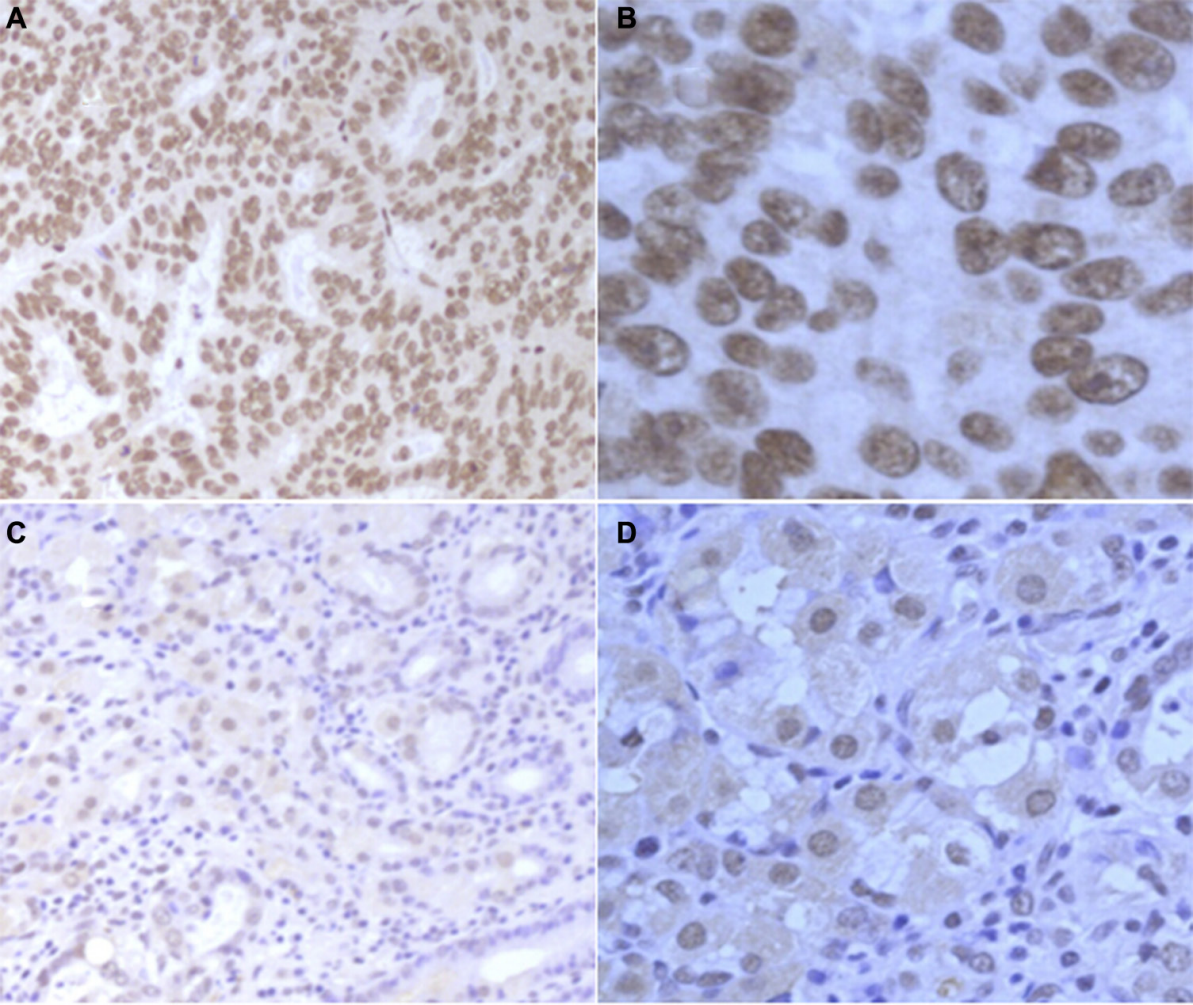
The protein expression of MDM4 in paraffin-embedded GTAC tissues and normal gastric tissues (**A**) MDM4 high expression in GTAC tissue (100×). (**B**) MDM4 high expression in GTAC tissue (400×). (**C**) MDM4 low expression in normal gastric tissue (100×). (**D**) MDM4 low expression in normal gastric tissue (400×).

Overall, 36.6% (123/336) of tumor sections and 15.2% (5/33) of the corresponding adjacent normal gastric tissue sections (*P* = 0.009) were classified as the MDM4 high expression. (Figure 3A–3D).

Statistical analyses were performed to investigate possible correlations between MDM4 expression and clinicopathological characteristics of patients. The analysis of 336 GTAC cases indicated that MDM4 expression was associated with age (*P* = 0.047), LNM (*P* < 0.001), pathological stage (*P* < 0.001), differentiation status (*P* = 0.001), and preoperative serum CA19-9 (*P* < 0.001) (Table 1).

**Table 1:** Correlation among MDM4 expression and the clinicopathological features of patients with GTAC

Variables	MDM4 (high) (123)	MDM4 (low) (213)	*P*
**Age (years)**			0.047
< 58	71 (57.7%)	99 (46.6%)	
≥ 58	52 (42.3%)	114 (53.6%)	
**Gender**			0.437
Male	84 (68.3%)	154 (72.3%)	
Female	39 (31.7%)	59 (27.7%)	
**Tumour size (cm)**			0.301
> 5	51(41.5%)	103 (48.4%)	
≤ 5	72 (58.5%)	109(51.6%)	
Borrmann type			0.402
0-II	35 (28.5%)	70 (32.9%)	
III-IV	88 (71.5%)	143 (67.1%)	
**Depth of invasion**			0.286
T1-2	33 (26.8%)	69 (32.4%)	
T3-4	90 (73.2%)	144 (67.6%)	
Lymph node metastasis			0.000
N^−^	18 (14.6%)	121 (56.8%)	
N^+^	105 (85.4%)	92 (43.2%)	
**Pathological Stage**			0.000
I	12 (9.8%)	57 (26.8%)	
II	37 (30.1%)	86 (40.4%)	
III	74 (60.2%)	70 (32.8%)	
Differentiation status			0.005
Differentiation	20 (16.3%)	64 (30.0%)	
Lack of differentiation	103 (83.7%)	149 (70.0%)	
**Preoperative serum CEA**			0.195
High	22 (17.9%)	51(23.9%)	
Normal	101 (82.1%)	162 (76.1%)	
**Preoperative serum CA19-9**			0.000
High	29 (23.6%)	18 (8.5%)	
Normal	94 (76.4%)	195 (91.5%)	

### Univariate and multivariate analyses of the predictor of clinical outcomes in GTAC

The univariate analysis showed a significant relationship between overall survival and tumor size [hazard ratio (HR) = 2.269; 95% confidence interval (95% CI) = 1.661–3.100; *P* < 0.001)], Borrmann type (HR = 2.810; 95% CI = 1.870–4.223; *P* < 0.001), depth of invasion (HR = 5.507; 95% CI = 3.329–9.108; *P* < 0.001), LNM (HR = 4.407; 95% CI = 2.990–6.494; *P* < 0.001), pathological stage (HR = 3.567; 95% CI = 2.593–4.906; *P* < 0.001), differentiation status (HR = 1.829; 95% CI = 1.224–2.732; *P* < 0.001), preoperative serum CA19-9 (HR = 2.691; 95% CI = 1.856–3.902; *P* < 0.001), and high MDM4 expression (HR = 1.943; 95% CI = 1.430–2.6413; *P* < 0.001). The multivariate analysis used the four significant parameters identified Borrmann type (HR = 1.719; 95% CI = 1.119–2.640; *P* = 0.013), depth of invasion (HR = 2.810; 95% CI = 1.551–5.092; *P* = 0.001), LNM (HR = 3.256; 95% CI = 1.913–5.541; *P* < 0.001), and preoperative serum CA19-9 (HR = 1.796; 95% CI = 1.208–2.671; *P* = 0.004) (Table 2).

**Table 2:** Univariate and multivariate analyses of the prognostic factors for TGAC patients

Variables	HR	Univariate 95% CI	*P*	HR	Multivariate 95% CI	*P*
**Age(years)**						
≥ 58 vs < 58	1.156	0.851–1.570	0.353			
**Gender**						
**Female vs Male**	1.294	0.914–1.833	0.147			
**Tumour size (cm)**						
> 5 vs ≤ 5	2.269	1.661–3.100	0.000	1.307	0.931–1.835	0.123
**Borrmann type**						
**III-IV vs 0-III**	**2.810**	**1.870–4.223**	**0.000**	**1.719**	**1.119–2.640**	**0.013**
**Depth of invasion**						
**T3-4 vs T1-2**	**5.507**	**3.329–9.108**	**0.000**	**2.810**	**1.551–5.092**	**0.001**
**Lymph node metastasis**						
**N+ vs N-**	**4.407**	**2.990–6.494**	**0.000**	**3.256**	**1.913–5.541**	**0.000**
**Pathological stage**						
**III vs I/II**	3.567	2.593–4.906	0.000	0.927	0.593–1.449	0.740
**Differentiation status**						
**Lack of differentiation vs Differentiation**	1.829	1.224–2.732	0.003	0.901	0.589–1.379	0.633
**Preoperative serum CEA**						
**High vs Normal**	1.135	0.787–1.637	0.498			
**Preoperative serum CA19-9**						
**High vs Normal**	**2.691**	**1.856–3.902**	**0.000**	**1.796**	**1.208–2.671**	**0.004**
**MDM4**						
**Positive vs Negative**	1.943	1.430–2.641	0.000	1.132	0.804–1.594	0.476

**Figure 4:**
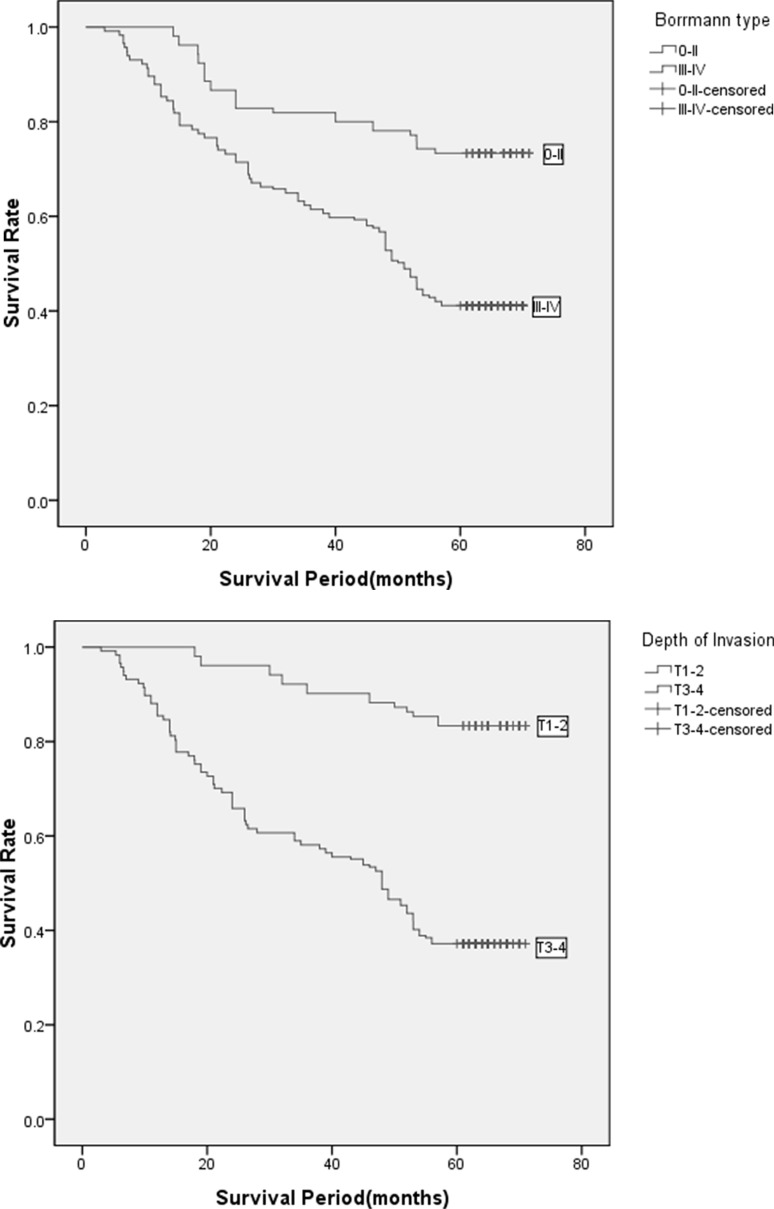

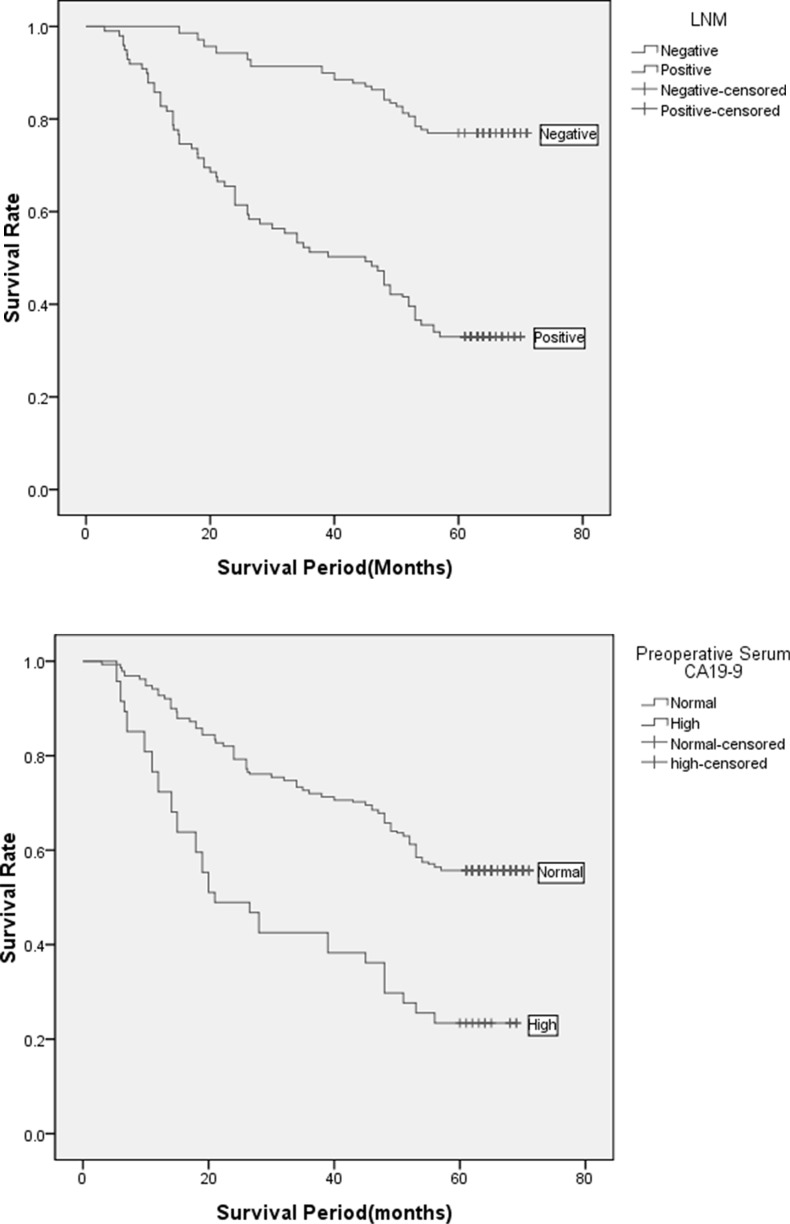
(**A**) Kaplan–Meier analysis of OS among GTAC patients based on Borrmann type. (**B**) Kaplan–Meier analysis of OS among GTAC patients based on Depth of Invasion. (**C**) Kaplan–Meier analysis of OS among GTAC patients based on the condition of lymph node metastasis. (**D**) Kaplan–Meier analysis of OS among GTAC patients based on the CA19-9 level of pre-operation.

The Kaplan–Meier analysis and log-rank test showed that patients with Borrmann types III–IV had a significantly shorter overall survival than those with Borrmann types 0–II (*P* = 0.013; Figure 4A). Patients with T3–4 had a significantly shorter overall survival than those with T1–2 (*P* = 0.001; Figure 4B). Patients with N^+^ had significantly shorter overall survival than those with N^−^ (*P* < 0.001; Figure 4C). Patients with high preoperative serum CA19-9 had significantly shorter overall survival than those with normal preoperative serum CA19-9 (*P* = 0.004; Figure 4D).

## DISCUSSION

LNM is one of the most important prognostic factors in patients with gastric cancer [5, 13]. The LNM status is the most important prognostic factor in patients with resectable gastric cancer, followed by pT staging, surgical complications, and distant metastasis [5]. Therefore, LNM status must be accurately assessed to predict the prognosis of patients and draft surgical plans. The 3D CT scan is commonly used in the clinical evaluation of patients with gastric cancer with LNM status based on the size of lymph nodes [6]. However, lymph node size is not a reliable indicator for LNM in patients with gastric cancer [14]. Preoperative accurate prediction of LNM status has special clinical significance for gastric cancer. To the best of our knowledge, this study is the first to demonstrate the correlation between LNM and the MDM4 expression in GTAC.

In this study, we investigated MDM4 expression status in GTAC at both mRNA and protein levels. MDM4 was significantly overexpressed in GTACN^+^. Furthermore, a high MDM4 expression level was significantly associated with age, lymph node metastatic status, pathological stage, differentiation status, and preoperative serum CA19-9. Gastric cancer in young adults was more likely associated with LNM [15, 16]. Lack of differentiation [17, 18] and high preoperative serum CA19-9 [19, 20] were associated with LNM in gastric cancer. High MDM4 expression level was significantly associated with age, LNM, pathological stage, differentiation status, and preoperative serum CA19-9. Our result was consistent with the previous research, which showed that high MDM4 expression was associated with LNM of GTAC. Ach et al. [21] showed that MDM4 aberrations were correlated with LNM of salivary gland cancer.

Borrmann type, depth of invasion, LNM (*P* < 0.001), and preoperative CA19-9 level (*P* = 0.009) were also independent factors influencing the prognosis of GTAC. Patients with Borrmann types III/IV often have a poor prognosis [22]. Particularly, lymph node and distant metastases are common in patients with Borrmann type IV [22–24]. Luo et al. [23] performed a meta-analysis of patients with gastric cancer of type IV Borrmann and poor tumor differentiation; the results indicated that these patients were prone to LNM, distant metastasis, serosal invasion, and lymph node invasion and had a poor prognosis. Our results showed that Borrmann type was an independent factor influencing GTAC prognosis. This result was consistent with that obtained by other researchers. Previous studies demonstrated that the depth of invasion was an independent prognostic indicator of GTAC [25, 26]. This study indicated that patients with T3–4 had significantly shorter overall survival than those with T1–2. The survival analysis showed that the depth of invasion was an independent prognostic indicator for GTAC. Such a finding was consistent with the previous research. LNM is the most important factor for the prognosis of patients with gastric cancer [27, 28]. In this study, high MDM4 expression was associated with LNM of GTAC. The univariate analysis showed that MDM4 expression influences the prognosis of GTAC. However, the multivariate analysis showed that the MDM4 expression was not an independent prognostic factor of GTAC. Accordingly, the influence of MDM4 on GTAC prognosis depended on whether it could lead to LNM of GTAC [28]. Increased CA19-9 level indicates poor GC prognosis [29, 30]. Yajima et al. [29] reported that CA19-9-producing gastric cancer had poor prognosis characterized by high preoperative serum CA19-9. Our findings showed that high preoperative serum CA19-9 was an independent prognostic factor for GTAC.

In conclusion, we showed that high MDM4 expression was associated with LNM of GTAC. The univariate analysis showed that MDM4 expression influenced the prognosis of GTAC, and the influence of overall prognosis relied on whether high MDM4 could lead to LNM. However, these findings need to be confirmed by a larger study.

## MATERIALS AND METHODS

### Patient and tissue specimens

Thirty pairs of fresh GTAC and corresponding normal mucosa tissue samples (more than 5 cm away from the GTAC edge) were obtained from patients between January 2015 and June 2015. And those samples were used for the Western blot analysis and the qRT-PCR analysis. A total of 336 paraffin-embedded tissues diagnosed with GTAC at the Department of Gastroenterologic Surgery, Harbin Medical University Cancer Hospital between January 2010 and December 2010, were used in the immunohistochemistry (IHC) analysis. Thirty-three matched normal gastric tissues were used as control samples. The 336 patients comprised of 238 men and 98 women aged 24–81 years old (mean age is 57.6 years old). None of the patients received preoperative anticancer treatment, and no patient had synchronous distant metastasis. Various clinicopathological parameters (i.e., age, gender, tumor size, Borrmann type, differentiation status, depth of invasion, LNM, pathological stage, preoperative serum CEA, and preoperative serum CA19-9) were obtained from histopathology records. The GTAC stage was described according to the 2010 tumor node metastasis classification of malignant tumors by the American Joint Committee on Cancer. The patients were followed-up until death or the last follow-up date (31 December 2015). All patients underwent complete follow-up, which ranged from 3 to 71 months. The patients provided written informed consents. An ethical approval was obtained from the Ethical Committee of Harbin Medical University Cancer Hospital.

### RNA extraction and qRT-PCR

Total RNAs were extracted from 30 pairs of fresh GTAC and adjacent normal gastric tissues by using E.Z.N.A.^®^ Total RNA Kit (Omega Biotek Store, USA). The qualified total RNAs were reversely transcribed into the first-strand cDNAs by using the PrimeScript® RT Reagent Kit (Takara). The forward primer for the MDM4 gene was 5′-CTAAGTCCTTAAGTGATGATACCGATGT-3′, and the reverse primer was 5′-AACTTTGAACAAT CTGAATACCAATCC-3′. The forward primer for the GAPDH gene was 5′-GGACCTGACCTGCCGTCTAG-3′the, and the reverse primer was 5′-GAGGAGTGGGT GTCGCTGTT-3′. The qRT-PCR was conducted using an ABI 7500 RT-PCR amplifier (Applied Biosystems, USA) to determine the expression pattern of MDM4 mRNA in each GTAC sample and paired adjacent normal gastric tissue. The qRT-PCR was then performed using the SYBR^®^ Premix Ex Taq™ II Kit (Takara) in a total volume of 20 μl. GAPDH was used as the reference gene. The relative gene expression levels were represented as ΔCt = Ct (gene) − Ct (reference). The fold change of gene expression was calculated using the 2^−ΔΔCt^ method. The experiments were repeated in triplicate.

### Western blot analysis

Total protein was isolated from 30 pairs of fresh GTAC and adjacent normal gastric tissues. An equal amount of protein from the tissues was separated through sodium dodecyl sulfate-polyacrylamide gel electrophoresis and transferred to a polyvinylidene membrane. The membranes were blocked with 5% fat-free milk in Tris-buffered saline containing 0.1% Tween-20 (TBST) for 1 h at room temperature. The membranes were then incubated overnight at 4°C with anti-MDM4 (1:1000, Abgent, USA) antibody or anti-GAPDH (1:1000, ZsBio, China). The membranes were washed three times with TBST buffer for 10 min and then incubated with the secondary antibody, anti-rabbit IgG (1:1000, ZsBio, China) or anti-mouse IgG (1:1000, ZsBio, China) at room temperature for 1 h. The membranes were subsequently washed with TBST three times. The immunoreactive bands were visualized using the ECL plus Western blot detection kit.

### IHC analysis

MDM4 expression in 336 GTAC and 33 normal gastric tissues was examined using IHC. The samples were fixed in formalin, embedded in paraffin, cut into 4 μm-thick sections, and mounted on silane-coated slides. Antigen was retrieved by immersing the samples in EDTA (pH 8.0) at high pressures for 2.5 min. The sections were then incubated in a humidified chamber at 4°C overnight with the primary antibody, mouse anti-MDM4 (diluted 1:250, Abcam, USA). The sections were incubated with biotinylated IgG secondary antibody (ZsBio, China) after washing three times in phosphate-buffered saline (PBS). The color was developed with 3,3′-diaminobenzidine DAB solution. The specificity of immunostaining was confirmed by obtaining negative controls through the replacement of the primary antibody with PBS. MDM4 expression was scored by multiplying the percentage of positive tumor cells and staining intensity. The percentage of the positive cells was initially scored into 0 (0%), 1 (1%–25%), 2 (25%–50%), 3 (50%–75%), and 4 (75%–100%). Thereafter, staining intensity was scored as follows: 0 (negative), 1 (weakly positive), 2 (moderately positive), and 3 (strongly positive). The immunostaining score, also known as staining index (SI), was calculated for each case by multiplying the percentage of positive cells with the staining intensity score, the resulting score is widely used in many malignant tumor studies [31, 32]. The immunohistochemistry scoring procedure was carried out to duplicate by two experienced pathologists to evaluate immunohistochemistry. The experienced pathologists did not know the clinicopathological information or the corresponding H&E slide. In the cases of scores with discrepancies, the results of immunohistochemistry were evaluated by additional pathologists until the consensus was reached. The obtained value ranged from 0 to 12. An optimal cut-off value was identified as follows: an SI score of six or higher was used to define tumors with high MDM4 protein expression level, and an SI score of less than six was used to indicate low expression levels.

### Statistical analysis

The statistical analysis was performed using the SPSS statistical software package (standard version 13.0; SPSS, Chicago, IL, USA). Student's *t*-test was used to analyze qRT-PCR data. The correlation between MDM4 expression and clinicopathological characteristics was analyzed using Pearson chi-squared test. The type of Cox regression model chosen was enter method. The survival curves were plotted using Kaplan–Meier method and then compared using the log-rank test.
